# Deficiency of Integrin *β*4 Results in Increased Lung Tissue Stiffness and Responds to Substrate Stiffness *via* Modulating RhoA Activity

**DOI:** 10.3389/fcell.2022.845440

**Published:** 2022-03-03

**Authors:** Yinxiu Chi, Yu Chen, Wang Jiang, Wenjie Huang, Mingxing Ouyang, Lei Liu, Yan Pan, Jingjing Li, Xiangping Qu, Huijun Liu, Chi Liu, Linhong Deng, Xiaoqun Qin, Yang Xiang

**Affiliations:** ^1^ School of Basic Medicine, Central South University, Changsha, China; ^2^ Changzhou Key Laboratory of Respiratory Medical Engineering, Institute of Biomedical Engineering and Health Sciences, Changzhou, China; ^3^ Longdong College, Qingyang, China; ^4^ Affiliated Liuzhou Maternity and Child Healthcare Hospital of Guangxi University of Science and Technology, Liuzhou, China

**Keywords:** integrin*β*4, lung stiffness, lysyl oxidase-like 4, collagen VI, RhoA

## Abstract

The interaction between extracellular matrix (ECM) and epithelial cells plays a key role in lung development. Our studies found that mice with conditional integrin *β*4 (ITGB4) knockout presented lung dysplasia and increased stiffness of lung tissues. In accordance with our previous studies regarding the functions of ITGB4 in bronchial epithelial cells (BECs), we hypothesize that the decreased ITGB4 expression during embryonic stage leads to abnormal ECM remodeling and increased tissue stiffness, thus impairing BECs motility and compromising lung development. In this study, we examined lung tissue stiffness in normal and ITGB4 deficiency mice using Atomic Force Microscopy (AFM), and demonstrated that ITGB4 deficiency resulted in increased lung tissue stiffness. The examination of ECM components collagen, elastin, and lysyl oxidase (LOX) family showed that the expression of type VI collagen, elastin and LOXL4 were significantly elevated in the ITGB4-deficiency mice, compared with those in normal groups. Airway epithelial cell migration and proliferation capacities on normal and stiff substrates were evaluated through video-microscopy and flow cytometry. The morphology of the cytoskeleton was detected by laser confocal microscopy, and RhoA activities were determined by fluorescence resonance energy transfer (FRET) microscopy. The results showed that migration and proliferation of ITGB4 deficiency cells were noticeably inhibited, along decreased cytoskeleton stabilization, and hampered RhoA activity, especially for cells cultured on the stiff substrate. These results suggest that decreased ITGB4 expression results in increased lung tissue stiffness and impairs the adaptation of bronchial epithelial cells to substrate stiffness, which may be related to the occurrence of broncho pulmonary dysplasia.

## 1 Introduction

Bronchopulmonary dysplasia (BPD) is a common chronic lung disease that inflicts upon premature infants due to complications arising from lung injury and mechanical ventilation. Converging evidence confirmed that children with BPD suffer from impaired lung structure, decreased pulmonary vascular growth, and compromised lung function, however, the exact pathogenesis of BPD remains to be clarified (Mushtaq, 2019; Duijts et al., 2020). One of the clinical characteristics of BPD is abnormal ECM stiffness, which stymies lung parenchymal growth (Mammoto et al., 2013). The extracellular substrate (ECM) is a dynamic, three-dimensional structure that is actively remodeled to promote the initiation, extension and formation of branches existent in all tissues (Chavan et al., 2012; Kim and Nelson, 2012). A sound ECM stiffness is essential to the regulation of lung morphogenesis (Caroline et al., 2014). By contrast, abnormal ECM stiffness leads to deregulated proliferation and migration of cells, thereby facilitating the pathological progression (Lu et al., 2011). The forming and shaping of the lung ECM have been widely concerned in the context of normal and aberrant lung alveolarization (Morrisey et al., 2013; Herriges and Morrisey, 2014; Surate Solaligue et al., 2017). Lysyl oxidase proteins (LOXs) are a class of amine oxidases mostly distributed in fibroblasts and smooth muscle cells contributing to the formation of ECM in a copper dependent manner (Bryan and D’Amore, 2007; Finney et al., 2014). The Lysyl-oxidase (LOXs) gene family is comprised of LOX and four LOX-like proteins (including LOXL1, LOXL2, LOXL3, and LOXL4), and play a key role in organ development via modulating ECM structure and mechanics through cross-linking of collagen and elastin (Smith-Mungo and Kagan, 1998; Lucero and Kagan, 2006; Barker et al., 2012). Previous research reported that infants with BPD show increased collagen abundance in the lung, abnormal collagen scaffolding and collagen fibers (Thibeault et al., 2003), as well as altered level of elastin (Bruce et al., 1985).

Integrins are a group of transmembrane receptors that mediate the interplay between cells and their extracellular environment. Integrins not only transduce extracellular signals to the actin cytoskeleton within the intracellular environment (outside-in signaling), but also transduce intracellular signals to the outside environment (inside-out signaling events), thereby converging on the regulation of cell signals, cell cycles, cell morphology, cell proliferation, cell differentiation and so forth (Sheppard, 2003). Our previous study found that the silencing of integrinβ4 (ITGB4) could delay the repair of bronchial epithelial cell injury, and undermine the ability of bronchial epithelial cells to resist oxidative injury (Liu et al., 2010a; Ryffel, 2012). Moreover, the aberrantly decreased expression of ITGB4 has been detected in the bronchial mucosa of animal models and asthmatic patients with specific variant site (Xiang et al., 2014). The high incidence of asthma in children suggests the plausible linkage between lung development and susceptibility to asthma.

Integrin beta 4 (ITGB4) is a structural adhesion molecule that functions to maintain the integrity of airway epithelial cells, owing to its distinct cytomembrane structural feature. The objective of the present study is to explore the mechanochemical mechanism of ITGB4 during lung development, in an attempt to explore the possibility of using ITGB4 as a novel target for the prevention and management of BPD. In the current study, we constructed a CCSP/TetO-Cre mouse model to delete ITGB4 specifically in bronchial epithelial cells of mice at the early stage of lung development. ITGB4 is engaged in several key physiologic processes and signaling pathways. Our data revealed that ITGB4-deficiency leads to aberrant tissue remodeling in the lung, and that the mechanical forces and chemical signal from the matrix mediate the motility of epithelial cells. Moreover, the perception and response of the matrix mechanical properties of ITGB4-loss cells were disparate from those of normal control cells. Taken together, these results consistently corroborate the role of ITGB4 as a new promising molecule mediating wound repair and ECM stiffness for airway epithelial cells.

## 2 Results

### 2.1 ITGB4 Deficiency Resulted in Abnormal Lung Structure

**FIGURE 1:**
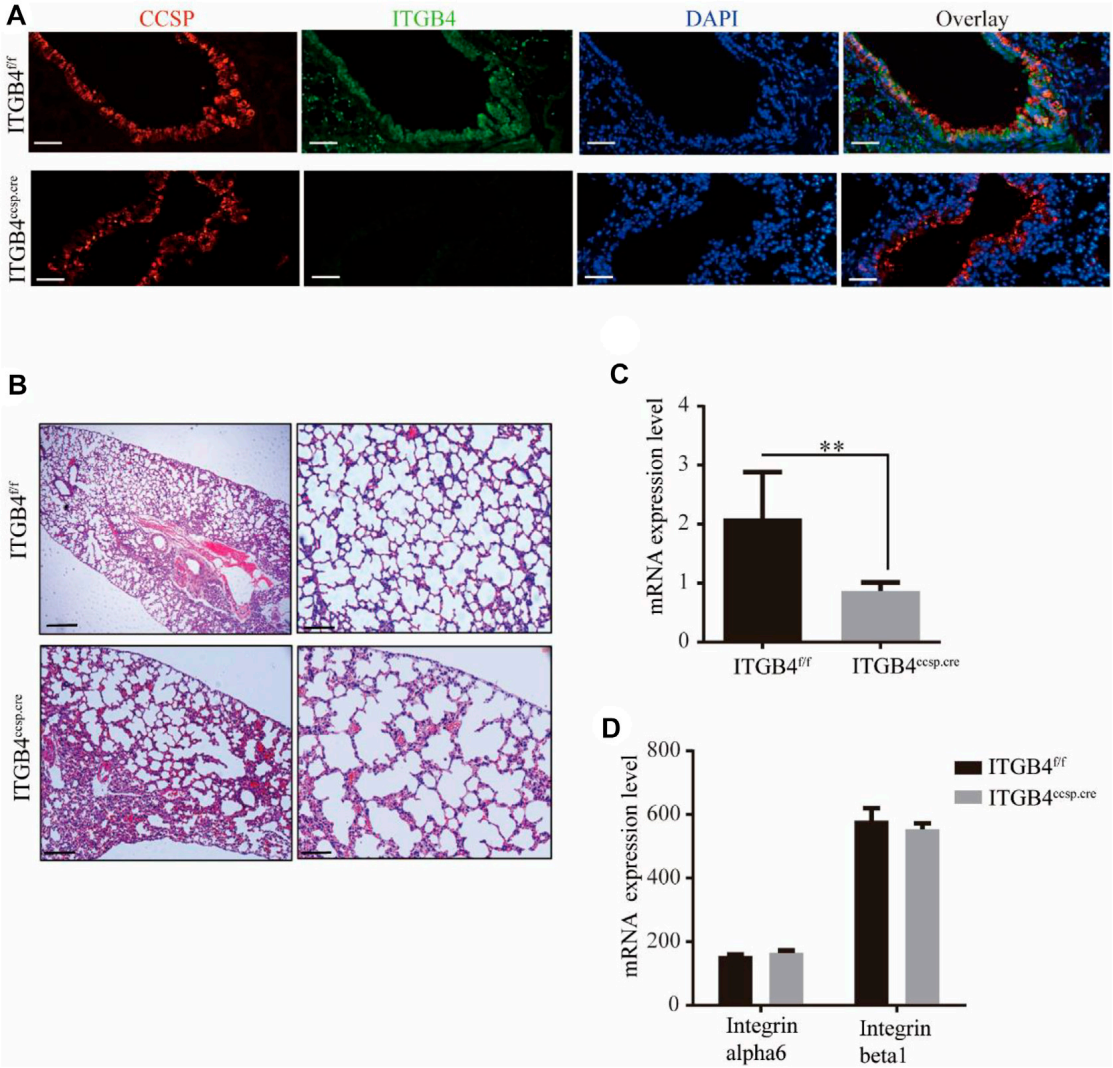
Silencing efficiency of ITGB4 and lung structure of ITGB4^f/f^ and ITGB4^ccsp.cre^ mice. **(A)** ITGB4 expression was detected by conducting immunofluorescence. CCSP (red) and ITGB4 (green) were performed in lung sections. DAPI was used to stain cell nuclei (blue).Scale bars: 50 μm **(B)** Dilated airspaces with thickened alveolar septa were seen in H&E-stained paraffin sections of P28 lungs. (Scale bars: 250 μm for left and 50 μm for right) **(C,D)** RT-PCR analysis of ITGB4, integrin alpha6, integrin beta1 mRNA expression levels in ITGB4f/fand ITGB4ccsp.cre mice. (**p < 0.01). Data are presented as mean ± SD.

To avoid the lethal effect of ITGB4 null on mice, we constructed a conditional ITGB4 deficiency model known as CCSP–rtTA^tg/−^/TetOCre^tg/−^/ITGB4^fl/fl^ (Ceteci et al., 2012; Hsu et al., 2014), in which ITGB4 was deleted only in airway epithelial cells, following the protocols elaborated in previous studies. Mice with selective airway epithelial cell deficiency of ITGB4 were generated by crossing ITGB4^fl/fl^ mice with CCSP–rtTA^tg/−^/TetO-Cre^tg/tg^ mice. In these mice, the reverse tetracycline responsive transactivator (rtTA) was expressed in airway epithelial cells under the control of CCSP promoter elements. In the presence of Dox, rtTA binds to the (tetO)7 CMV promoter, which activates Cre-recombinase expression and deletes the ITGB4 gene (Liu et al., 2018). *β*4^f/f^ was referred as the control group, and the *β*4^ccsp.cre^ was referred as the experiment group. To examine the expression of ITGB4 in airway epithelial cells of mice, we performed RT-PCR and dual immunofluorescence staining. As shown in Figures 1A–C, ITGB4 was selectively deleted in the airway epithelium. The examination of mRNA levels of ITGB4-related genes showed that the expression levels of integrin *α*6 (a ligand for integrin *β*4) and integrin *β*1 were not significantly affected by the knockout of ITGB4, compared to those in *β*4^f/f^ mice (Figure 1D). Histological examination (H and E staining) was conducted to examine alveolar structure at postnatal day 28 (P28) to examine the impact of ITGB4 deficiency. The results of H&E staining showed clear alveolar structure in β4^f/f^ group (Figures 1A,B). In contrast, the *β*4^ccsp.cre^group revealed dilated airspaces surrounded by thickened alveolar walls, the lack of alveolar septation, and large alveolar size. (Figure 1B–D). Such morphological disparities between the two groups suggest that epithelial-specific knockout of ITGB4 leads to lung dysplasia.

### 2.2 ITGB4 Deficiency Resulted in Increased Lung Tissue Stiffness

The mechanical properties of extracellular matrix regulate a variety of key events in pathogenesis, fibrosis, and organ development. As a distinct mechanical property, the stiffness of extracellular matrix is the primary determinant of cell and tissue behavior, and is implicated in lung development.

**FIGURE 2:**
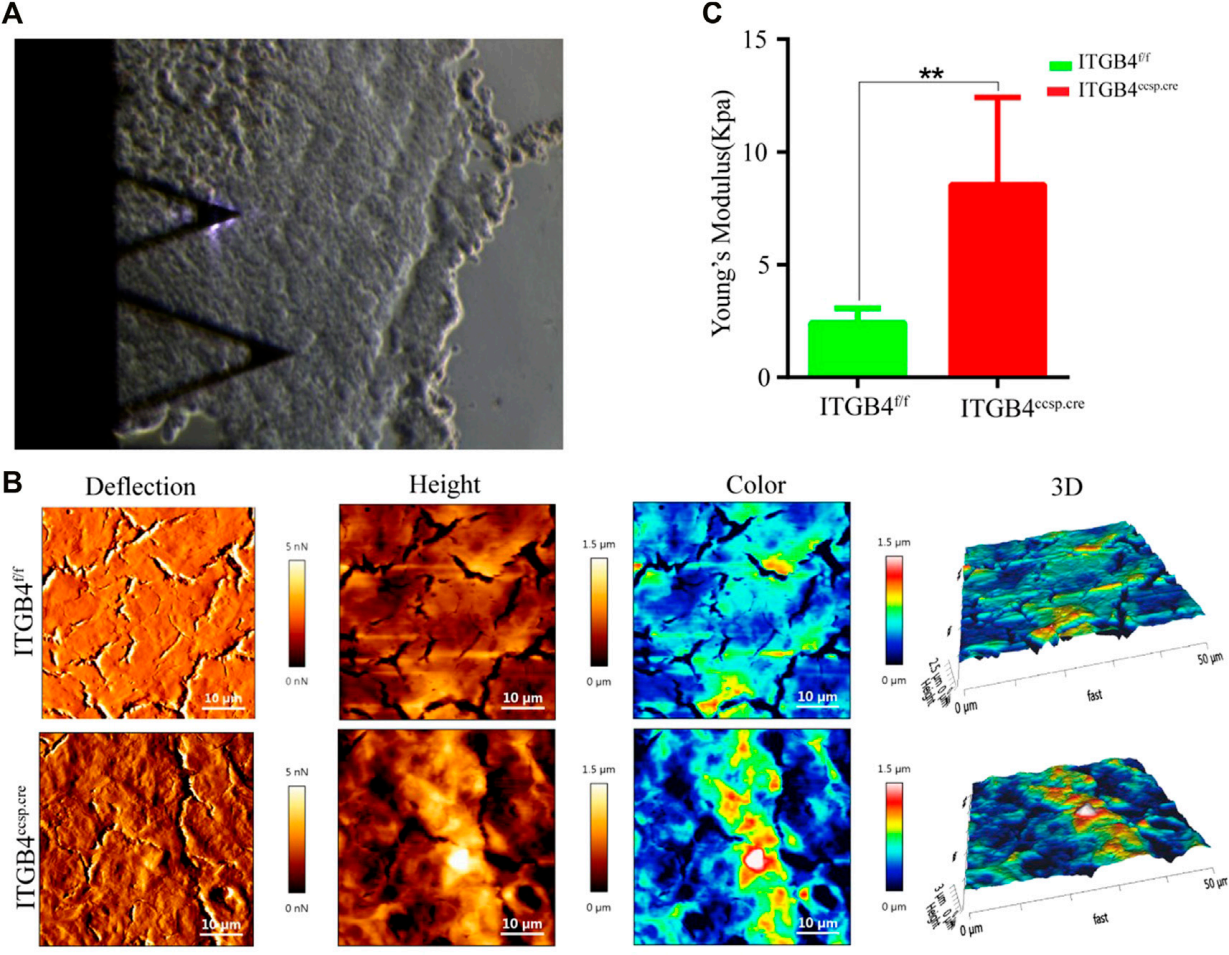
The morphology and mechanical properties of ITGB4^f/f^ and ITGB4^ccsp.cre^mice was detected by using AFM. **(A)** Optical microscopy image of the lung section with the AFM tip in contact. **(B)** Representative lung tissue surface images in AFM contact mode (deflection image; height image; color image and 3D image). **(C)** lung tissue stiffness or Young’s modulus were calculated by fitting the force-indentation curve to Hertz mode. (*n* = 3 independent experiments, **p < 0.01). Data are presented as mean ± SD.

In the study, Atomic Force Microscopy (AFM) was applied to observe the morphological characterization of lung tissues and to examine the possible effect of ITGB4 deficiency on changes of lung mechanical properties. Precision-cut lung tissue slices were prepared for AFM measurement. The morphological characteristics of lung tissue from β4^f/f^ group and *β*4^ccsp.cre^ group were shown in Figure 2B. Apparently, *β*4^f/f^ mouse lung had smooth surface with well-organized cells, while the surface of *β*4^ccsp.cre^ mouse lung tissue was rough. The results of AFM also verified increased roughness of *β*4^ccsp.cre^ mouse lung tissue in comparison with that of *β*4^f/f^ mouse, suggesting the occurrence of ECM remodeling in *β*4^ccsp.cre^ mice, possibly by collagen rearrangement. Lung tissue stiffness was measured by in accordance with the average value of Young’s modulus, which was calculated by fitting the force-indentation curve to Hertz model. The results showed that the stiffness of *β*4^f/f^ mouse lung tissue was significantly lower than that of *β*4^ccsp.cre^ mouse (2.41 ± 0.66 kPa vs. 8.54 ± 3.87 kPa, *p < 0.01*) (Figure 2C).

### 2.3 ITGB4 Deficiency Resulted in Altered Spatial Structure and Distribution of Col VI, Elastin and loxl4 in *β*4^ccsp.cre^ Mice

**FIGURE 3:**
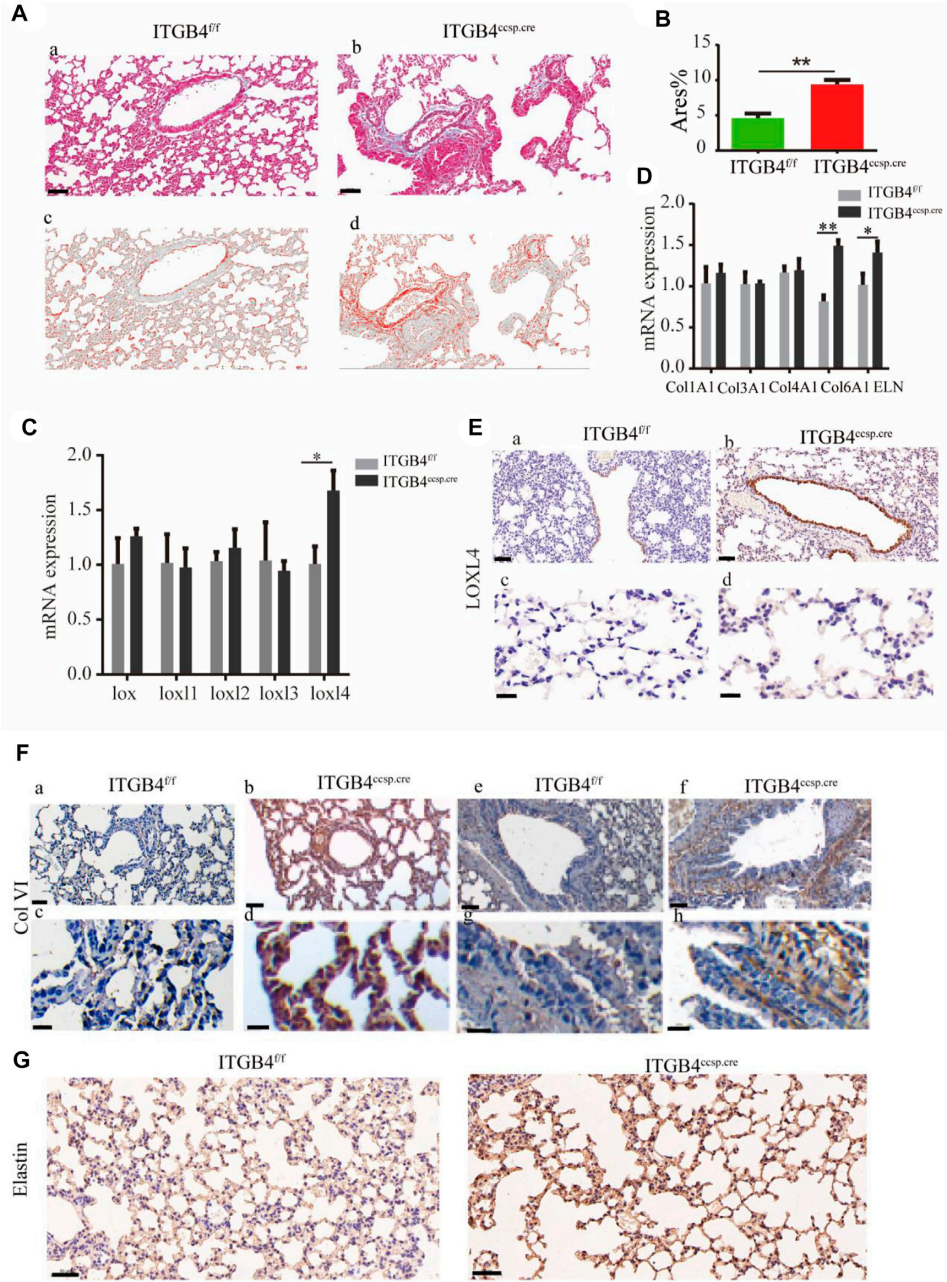
The expression of collagens, elastin and LOXs between ITGB4^f/f^ and ITGB4^ccsp.cre^ mice. **(A,B)** Masson’s Trichrome stain image (a, b) and ImageJ Imaging Software-generated image (c, d) to calculate Area % of collagen (Red). Scale bars: 50 μm. **(C,D)** The mRNA expression levels of collagens, elastin and LOXs were detected by RT-PCR. **(E)** Immunostaining for LOXL4 in airway epithelium (a,b) and alveolar septa (c,d) in ITGB4^f/f^and ITGB4^ccsp.cre^lungs. Scale bars: 50 μm for (a, b);20 μm for (c, d). **(F)** Immunostaining for COLVI in pulmonary alveoli (a–d) and in airway epithelium (e–h) in ITGB4^f/f^ and ITGB4^ccsp.cre^lungs. Scale bars: 200 μm for (a, b, e, f); 50 μm for (c, d, g, h). **(G)** Immunostaining for elastin in pulmonary alveoli. Scale bars: 20 μm. (*n* = 3 independent experiments, *p < 0.05, **p < 0.01). Data are presented as mean ± SD.

The dynamics of lung ECM remodeling can be induced by changed composition of ECM. Specifically, the increased collagen levels and altered collagen cross-linking could contribute to enhanced tissue stiffness (Brinckmann et al., 2001; Chen et al., 2015). We quantified the collagen deposition in the lung tissue by performing Masson staining (Figures 3A,B) to observe the expression pattern of various components of ECM in lung tissue. Subsequently, qRT-PCR assay was carried out to examine mRNA levels of collagens and LOXs in mouse lungs. The results showed that the levels of Col VI, elastin and LOXL4 were markedly elevated in *β*4^ccsp.cre^ mice (Figures 3C,D).

**FIGURE 4:**
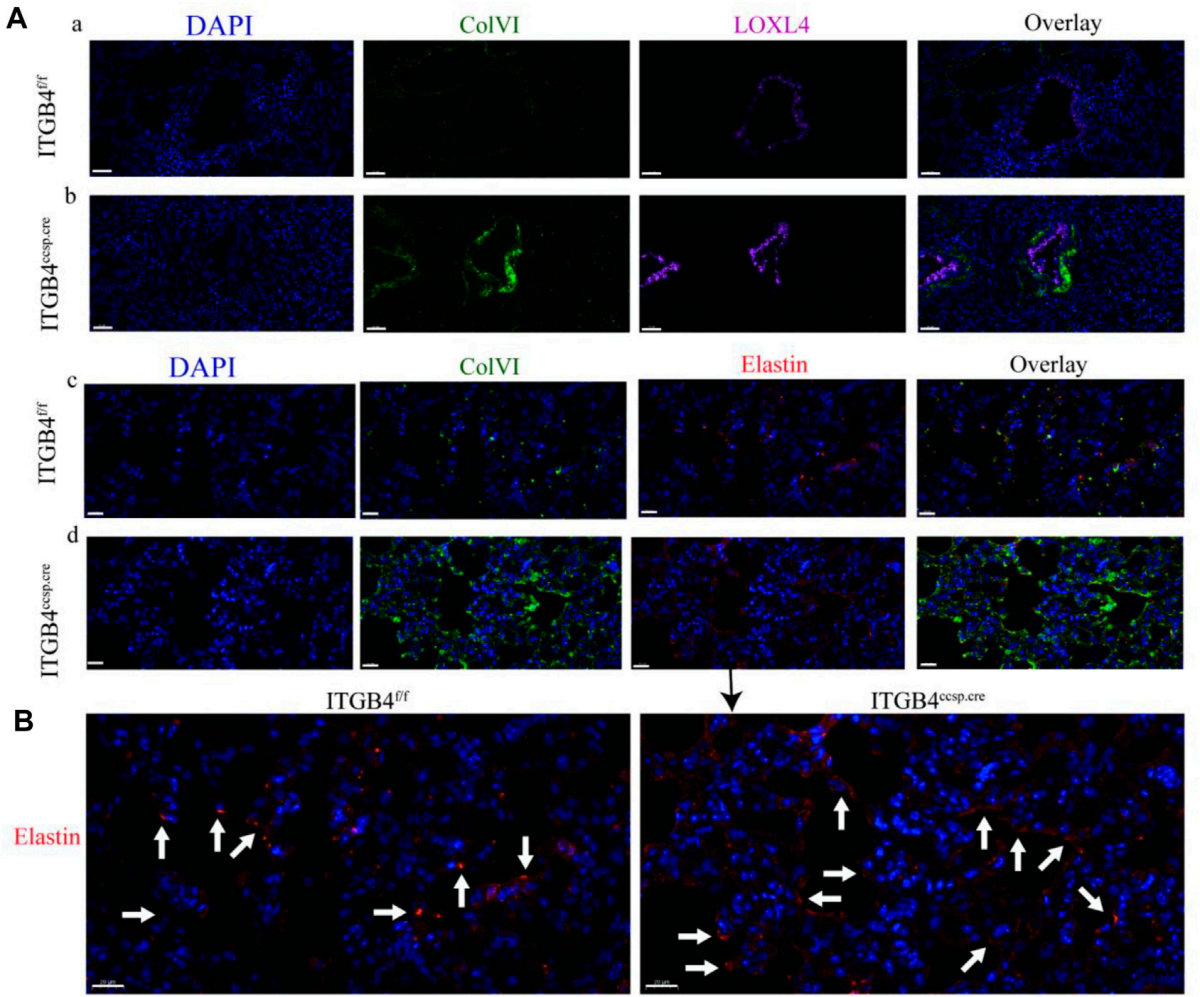
The expression of LOXL4,ColⅥ and elastin between ITGB4^f/f^ and. ITGB4^ccsp.cre^ mice **(A)** Immunofluorescence images of Col VI (green) and LOXL4 (pink) in the airway epithelium (a, b) and the Col VI (green) and elastin (red) in alveoli (c, d). Scale bars: 50 μm. **(B)** Immunofluorescence images of elastin in the in alveoli. Scale bars: 20 μm. Lung elastin accumulation (arrows) was distinctly greater in the ITGB4^ccsp.cre^ lungs.

The results of immunohistochemical experiment showed that the expression of LOXL4 (Figure 3E) and Col VI (Figure 3F) was significantly up-regulated in the airway and alveolar tissue of *β*4^ccsp.cre^ mice. The expression of elastin was also increased in alveoli of *β*4^ccsp.cre^ mice (Figure 3G). Immunofluorescence (IF) results showed basal expression of LOXL4 in epithelial cells of *β*4^f/f^ mice, whereas much higher levels in those of *β*4^ccsp.cre^ mice (Figure 4Aa,b). Moreover, sub-epithelial deposition of Col VI was found in β4^ccsp.cre^ mice. A remarkable variation in the distribution of elastin deposition in the alveolar region could be observed between *β*4^f/f^and *β*4^ccsp.cre^ mice (Figures 4Ac,d). As shown in Figure 4B, elastin was expressed mainly at the tips of alveolar septa in the lungs of *β*4^f/f^ mice, whereas elastic fibers were prominently distributed throughout the walls of respiratory units in the lungs of *β*4^ccsp.cre^ mice. These results suggest that ITGB4 loss-of-function induces ECM remodeling of lung tissue, thereby increasing tissue stiffness.

### 2.4 The Role of ITGB4 in Airway Epithelial Cell (16HBE14o-Cells) Response to Substrate Stiffness *in vitro*

As mentioned above, ITGB4 deficiency results in aberrant tissue remodeling in the lung. Our *in vivo* results (Figure 4) support ITGB4 deficiency leading to ECM remodeling and lung tissue stiffening. In the following part, we analyzed the biological role of ITGB4 in regulating the motility of airway epithelial cell in response to alerted ECM stiffness.

#### 2.4.1 ITGB4 Expression Was Reduced on the Stiff Substrate

**FIGURE 5:**
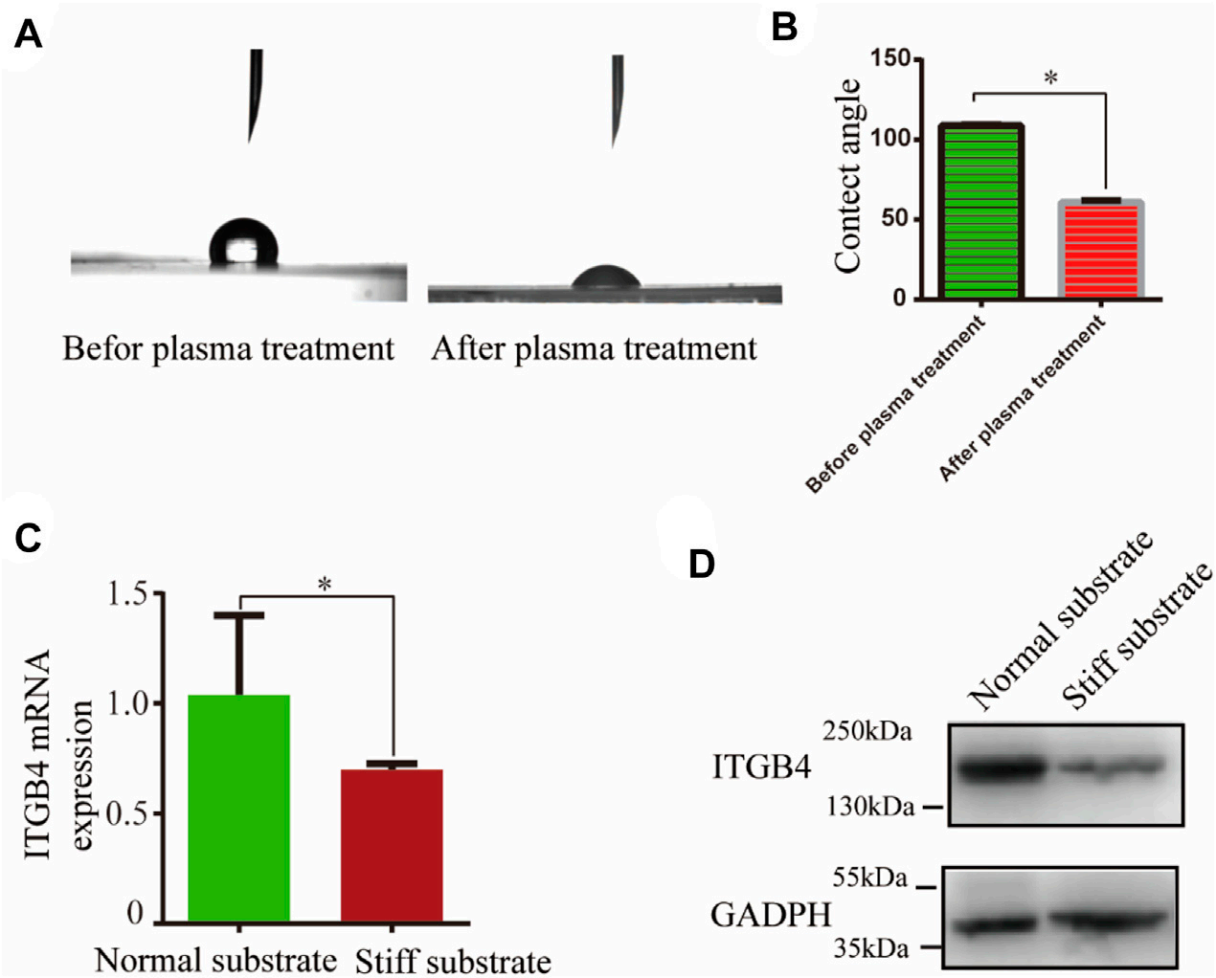
Normal and stiff substrate models were constructed *in vitro*. **(A,B)** Detection of contact angle and the contact angle before and after plasma treatment **(C,D)** ITGB4 expression of 16HBE14o-cells on the normal and stiff substrate by RT-PCR and western blot. (*n* = 3 independent experiments, *p < 0.05, **p < 0.01). Data are presented as mean ± SD.

While ITGB4 deficiency results in aberrant tissue remodeling in the lung, mechanical and chemical signal from the matrix could influence the motility of epithelial cells. To determine whether ITGB4 mediates cellular motility in a substrate stiffness-dependent manner, we fabricated two types of PDMS substrates with different elastic moduli. AFM test showed the PDMS stiffness at 2 and 9 kPa, respectively, to mimic the lung tissue stiffness of normal and ITGB4-silent mice. Plasma treatment was performed to characterize the coating effect. A contact angle below 90° is related to a hydrophilic substrate, whereas a contact angle value above 90° is related to a hydrophobic substrate, according to the previously described protocol (Pandey et al., 2019). In this study, the contact angle was 108.66 ± 0.44° before plasma treatment and 60.6 ± 1.3° after plasma treatment, respectively (Figures 5A,B). To facilitate cellular proliferation, Matrigel was coated on the normal substates and stiff substrate after plasma treatment. Afterwards, 16HBE14o-cells were cultured on the constructed normal and stiff substrate. The results of RT-PCR and western blot analysis consistently showed that ITGB4 mRNA and protein expression were significantly down-regulated in cells cultured on the stiff substrate than cells cultured on normal substrate (*p < 0.05, Figures 5C,D).

#### 2.4.2 ITGB4 Deficiency Resulted in Impaired Cell Migration and Proliferation, Especially on the Stiff Substrate

##### 2.4.2.1 Wound Healing Assay

**FIGURE 6:**
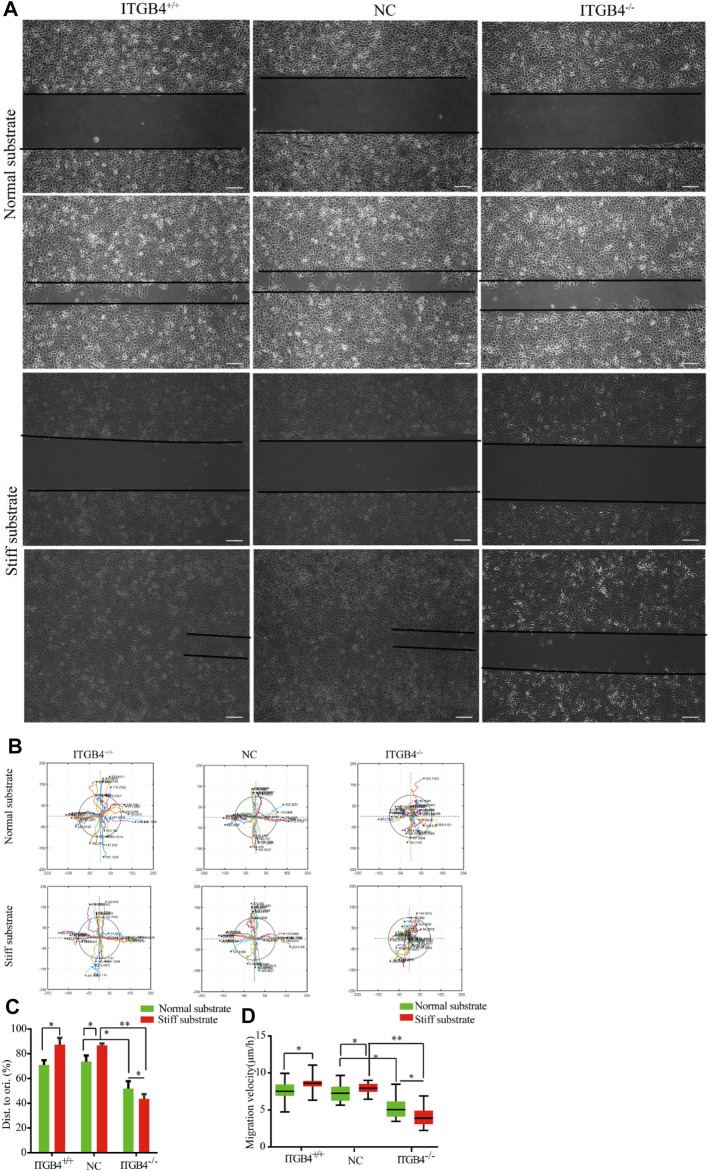
Cells wound repair on the normal and stiff substrates. **(A)** Representative images of wound-healing of cells on the normal and stiff substrates. **(B)** Track migration of cells cultured on the normal and stiff matrix, monitored for 24 h (*n* = 40). Tracks were recorded in the *x*/*y* direction. Each value indicates the total distance travelled by the cells in 24 h; the line colors reveal the trajectory of different cells; the colors were randomly assigned. **(C)** Quantification of the rate of repair at 24 h post-injury (relative to 0 h, *n* = 40). **(D)** Box-and-whisker plot of cell migration velocity (*n* = 40). (*n* = 3 independent experiments, *p < 0.05; **p < 0.01). Data are presented as mean ± SD.

During lung development, migration of epithelial cells to the interstitium regularly and directionally to contributes to the formation of pulmonary branch morphology. During embryogenesis, the population of migrating cells relies on mechanical response to external stimuli and signal transduction. To examine whether cell migration was subject to various stiffness of substrates, we cultured 16HBE14o-cells on the normal and stiff substrate, then divided the cells into the following subgroups: normal control groups (ITGB4^+/+^), ITGB4 -knockdown groups (ITGB4^−/−^) and nonsense-RNA groups (NC). A scratch wound assay was conducted to examine the migratory properties of the cells; a faster wound repair of cells was observed on the stiff substrate, rather than on the normal substate (*p < 0.05). However, in the event of ITGB4 knockdown, cell migration became significantly slower both on both normal (*p < 0.05) and stiff substrates than the control group. Of note, the migration of ITGB4^−/−^ cells was dramatically retarded on the stiff substrate (**p < 0.01) (Figures 6A,D).

##### 2.4.2.2 Tracking of Cell Migration

Time-lapse microscopy was performed to analyze the individual cell movements. To quantify these differences in cell migration, individual cells were tracked for 24 h, and the representative tracks of 40 cells were recorded. Normal cells showed significantly fast migration velocity and longer distance of travelling. No significant difference could be found in terms of directionality between cells on normal and stiff substrates. In stark contrast, the migration of ITGB4^−/−^ cells became slower and exhibited random directions. Such migratory pattern was distinctly prevalent for cells cultured on the stiff substrate, along with disarranged array of cells and less distance of migration from the starting point (Figures 6B,C).

##### 2.4.2.3 Cell Proliferation Assay

**FIGURE 7:**
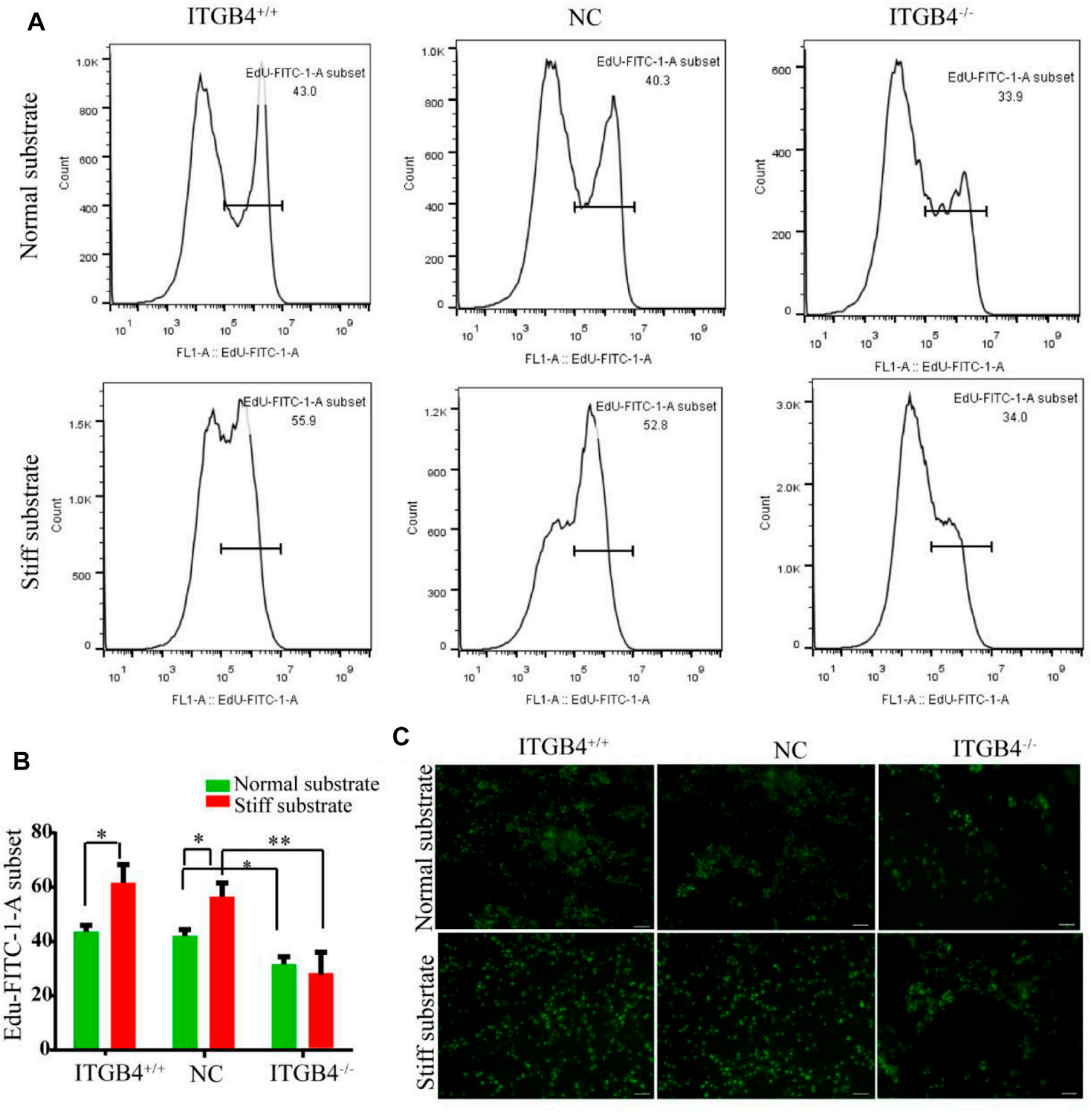
Proliferation of cells on the normal and stiff substrates. **(A)** Proliferation of cells was determined by flow cytometry. **(B)** Flow cytometry results were quantified. **(C)** The proliferation of cells was detected by using the EdU kit. (*n* = 3 independent experiments, *p < 0.05; **p < 0.01). Data are presented as mean ± SD.

Subsequently, to determine whether the ITGB4 may regulate the proliferation ability of cells, we performed flow cytometry by using commercialized E-dU kit. The results indicated stronger proliferative capacity of cells cultured on the stiff substrate than on the normal substrate (Figure 7A,B). The cells cultured on the normal substrate were mostly concentrated in clusters, while those on the stiff substrate evenly dispersed. Moreover, the cells cultured on the stiff substrate exhibited more potent capacity of proliferation than those cultured on the normal substrate. In the event of ITGB4 knockdown, the proliferation of cells cultured on both two types of substrates were weakened, such pattern was most manifested for cells cultured on the stiff substrate (Figure 7C). These findings suggested that ITGB4 deficiency resulted in impaired potential of cell migration and proliferation, especially on the stiff substrate.

#### 2.4.3 ITGB4 Deficiency Resulted in Abnormal Cytoskeletal Assembly, Especially on the Stiff Substrate

**FIGURE 8:**
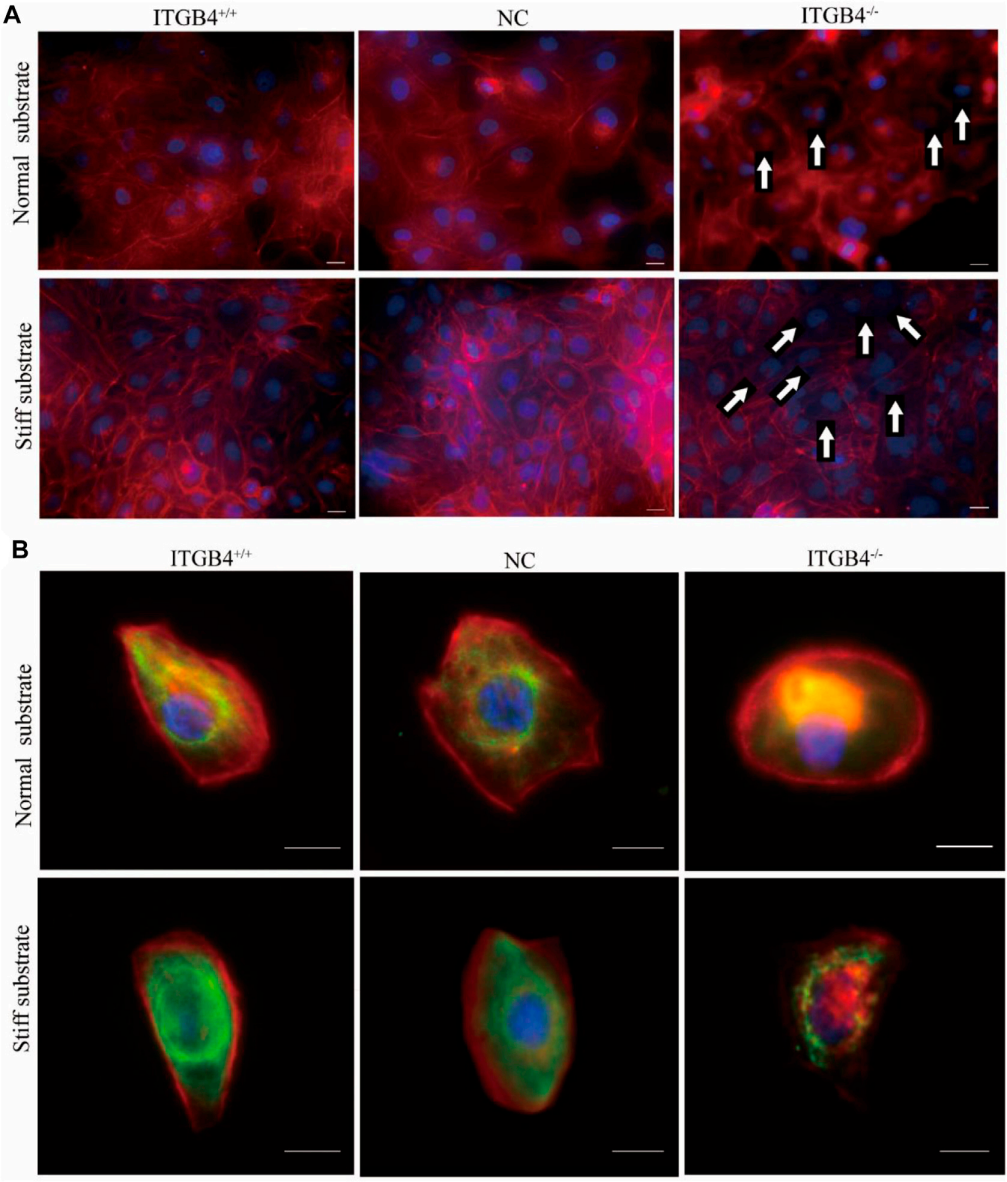
Cytoskeletal morphology of cells on the normal and stiff substrates. **(A)** Confocal images of cells immunostained for F-actin. (F-actin staining with Phalloidin, Nuclear staining with DAPI) (The arrow shows that F-actin aggregates in the perinuclear area of cells cultured on normal substrate, and that F-actin mostly vanished in the cells culture on stiff substrate). **(B)** Confocal image of cells dual-labeled for actin and vimentin. Red fluorescence indicates F-actin, and green fluorescence indicates vimentin.

Substrate stiffness can influence cell movements by regulating the assembly of the cytoskeleton. We performed immunofluorescence experiment to examine the cytoskeletons of cells cultured on the normal and stiff substrate, and to specify the biological role of ITGB4 on cytoskeletal assembly. For cells cultured on normal substrate, some microfilaments were localized in the cytoplasm, along with signs of perinuclear accumulation. However, the cytoskeleton of cells cultured on stiff substrate spread well, with clearly visible microfilaments. Moreover, the cells exhibited elevated expression of vimentin, which was well-distributed on the stiff substrate. ITGB4^−/−^ cells cultured on normal and stiff substrates underwent abnormal skeletal organization. Specifically, F-actin microfilaments decreased and were accumulated accumulate excessively in the perinuclear fraction of ITGB4^−/−^ cells cultured on the normal substrate, along with notably reduced expression of vimentin. ITGB4^−/−^ cells cultured on the stiff substrate exhibited abnormal reorganization of cytoskeleton, dissolved microfilament structure, and negligible expression of vimentin (Figures 8A,B). These data indicate that the cytoskeleton of ITGB4^−/−^ cells, unlike normal control cells, were incapable of adapting to the stiff substrate, as illustrated by the disarranged cytoskeleton.

#### 2.4.4 ITGB4 Deficiency Induced Decreased RhoA Activity, Especially on the Stiff Substrate

**FIGURE 9:**
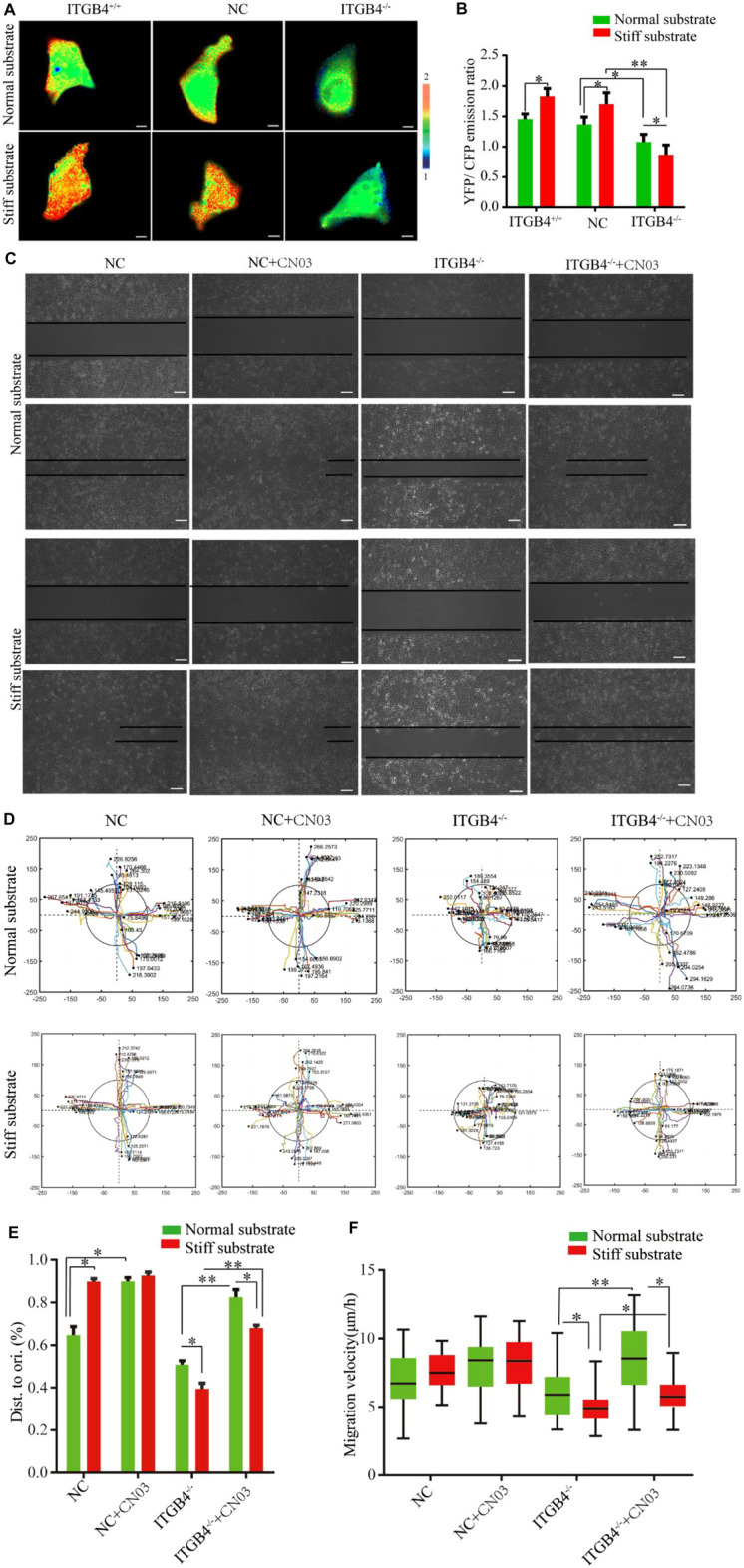
RhoA activity and migration of cells. **(A, B)** Images of the emission ratios of YFP/CFP-based RhoA biosensor in cells on normal and stiff substrate. Cells are shown on the left, the right panels show the emission ratios of the YFP/CFP-based RhoA biosensors **(C)** Representative images of wound-healing response for NC and ITGB4. ^−/−^ cells on normal and stiff substrate before and after CN03 treatment. **(D)** Track migration of NC and ITGB4^−/−^ cells on the normal and stiff substrate before and after CN03 treatment, monitored for 24 h (*n* = 40). Tracks were recorded in the *x*/*y* directions. **(E)** Quantitation of the rate of repair at 24 h post-injury (relative to 0 h, *n* = 40). **(F)** Box-and-whisker plot of cell migration velocity (*n* = 40). (*n* = 3 independent experiments, *p < 0.05; **p < 0.01). Data are presented as mean ± SD.

Integrin signaling can activate RhoA, which is deeply involved in the reorganization of cell cytoskeleton (Mégarbané et al., 2020; Noorman et al., 2009). Here, the activation levels of RhoA in cells on the normal and stiff substrates were evaluated by using a unimolecular effector-based RhoA FRET biosensor (Ouyang et al., 2008; Seong et al., 2011). The results found notably higher level of RhoA in normal cells cultured on the stiff substrates. However, RhoA activity was reduced in ITGB4^−/−^ cells cultured on both normal and stiff substrates. Of note, ITGB4^−/−^ cells cultured on the stiff substrate had a lower RhoA activity than those on the normal substrate (Figures 9A,B). To confirm the association between RhoA activation and cell migration disorders induced by ITGB4-silencing, the cells were treated with CN03, a specific agonist of RhoA (Xie et al., 2020). The results suggested that cell migration was strengthened on the normal substrate with the intervention of CN03, yet it was mildly changed on the stiff substrate. For ITGB4^−/−^ cells, the migratory capacity increased to a greater extent after being co-cultured with of CN03 on the normal substrate than those on the stiff substrate (Figures 9C–E). ITGB4^−/−^ cells cultured on normal substrate showed an accelerated speed of wound healing (Figure 9F).

## 3 Discussion

The unique molecular structure and intracellular signal transduction mechanism make ITGB4 play a vital role in cell-ECM signal interaction (Sheppard, 2003). In the present study, we constructed a CCSP/TetO-Cre mouse model to conditionally knockdown ITGB4 in the airway epithelium, and found defective lung alveolar development and toughened tissue stiffness in the animal model. Integrin-ligand interaction plays a part in regulating a myriad of cellular events, including adhesion, migration, differentiation, and even biomedical device integration (Karimi et al., 2018). In this study, we examined the effect of integrin *β*4-loss on the expression of integrin *α*6 and integrin *β*1, both of which are involved in airway epithelial cell (Colburn and Jones, 2017). The negligibly altered level of integrin *β*1 in integrin *β*4-loss group indicated that integrin *β*1 less important in lung development disorder induced by integrin *β*4-knockdown.

Recent studies have emphasized that mechanical factors, including substrate stiffness, can mediate cell motility and effect morphological change, thus contributing to a key process for regulating tissue structure and size (Cai et al., 2014). The development of the lung is a process in which the epithelial structure continues to grow into the lung interstitium. On the one hand, the cells perceive the matrix through cytoskeleton tension and matrix interactions, maintaining the balance of cell mechanics to drive cell deformation and rearrangement. On the other hand, the continuous differentiation or rearrangement of the cells continuously adjust ECM components to change the composition and morphology of the ECM (Cheng et al., 2016), thereby establishing a feedback mechanism between the cell and the ECM underlying the quick response of the cells to subtle environmental changes and to adjust its behavior accordingly (Bonnans et al., 2014). While the level of integrin *β*4 was lower in cells cultured on stiff substrate, the normal alveolar epithelial cells (AECs) are capable of adapting to the substrate. A series of unfavorable variations, including higher tissue stiffness, were most predominant in ITGB4-loss cells cultured on the stiff substrate.

To date, the mechanical conversion of the structural characteristics of the matrix and the rigidity signal transduction into intracellular signals is still not clear. The interruption of normal homeostasis between ECM production and degradation changes could result in significant stiffness changes in the ECM. The occurrence of collagen rearrangement and deposition is a key indicator of increased tissue stiffness (Provenzano et al., 2009), and the activation of LOXs leads to tissue stiffening by crosslinking collagen and elastin fibers (Finney et al., 2014). Lung manifests unique ECM composition and function. Collagen type I, III and elastin jointly form the bulk of ECM in the lung; Laminin and collagen IV are the major components of basement membrane; type VI collagen is largely distributed in or near the basement membrane. In the current study, we found that the mRNA levels of COL VI, elastin and LOXL4 were notably increased in lung tissue of *β*4^ccsp.cre^ mice. COL VI is distributed in the lamina propria under the epithelial basement membrane to connect the components of the basement membrane (Bonaldo, 1998; Bober et al., 2010; Dassah et al., 2014). It has been reported that lung alveolarization defect is attributable to impaired elastin deposition (Bruns et al., 1986; Malmström et al., 2003; Abdillahi et al., 2015; Theocharidis et al., 2016). Our data established a close correlation between the changing patterns of Col VL, elastin and loxl4 with lung tissue stiffness in ITGB4-silencing mice.

Our experimental results showed that ITGB4-loss led to increased lung tissue stiffness. To examine the possible influence of higher tissue stiffness on cell migration and proliferation, PDMS substrates with different stiffness were made to simulate normal lung tissue stiffness at 2 Kpa and ITGB4-deficiency lung tissue stiffness at 9 KPa, respectively. The results showed augmented migration and proliferation capacities of cells cultured on the stiff substrate, which is consistent with that of previous literature (Sunyer et al., 2016; Zhao et al., 2018a). Meanwhile, cell trajectory analysis showed no differences between the normal control cells cultured on normal and stiff substrate. However, the migration and proliferation abilities of ITGB4-deficiency cells cultured on the normal substrate were stymied. Interestingly, the migration and proliferation of the cells on the stiff substrate were diminished to the greatest extent.

Considering that cells’ response to the stiff substrate and cytoskeleton remodeling were closely associated with motility and proliferation (Colburn and Jones, 2017), we observed the structural changes in cytoskeleton. It was found that microfilaments were localized to the perinuclear region, and distributed along the edge of the cells on the normal substrate. In contrast, microfilaments were distributed throughout the cytoplasm of the cells cultured on the stiff substrate. Of note, the distribution of microfilaments of ITGB4-knockdown cells cultured on normal substrate remained unchanged, mostly in the perinuclear regions, whereas the actin microfilament structure of ITGB4-knockdown cells on the stiff substrate appeared reduced or even lost. Moreover, vimentin expression was fragmented and severely down-regulated and appeared “clump-like” change of staining. The above results suggest that cells on the normal substrate could better adapt to cytoskeleton remodeling, and that the motility and proliferation ability of cells were less damaged compared to those on the stiff substrate.

RhoA, a small GTPase, is an essential modifier of actin cytoskeleton and reorganization in the downstream of integrin engagement (Huveneers and Danen, 2009). Evidence demonstrated that substrate stiffness regulated the migratory and invasive ability of cells via RhoA/ROCK pathway and that enhanced RhoA activity is indicative of a variety of diseases (including IPF, Idiopathic pulmonary fibrosis) (Zhao et al., 2018b). Our data showed that RhoA activity was higher in cells cultured on the stiff substrate, whereas cell migration was mildly enhanced following the addition of RhoA activator CN03, which could mostly be attributed to the saturated rate of wound healing. However, following the constitutive activation of RhoA *via* CN03 treatment, we observed improved migratory ability of ITGB4^−/−^ cells on normal and stiff substrates, especially for the cells cultured on normal substrate. Such disparity could be justified by the down-regulated expression of RhoA in ITGB4^−/−^cell on the stiff substrate, which led to weakened compensation effect of CN03.

In summary, ITGB4 deficiency leads to increase lung tissue stiffness *in vivo*, ECM remodeling, significantly decreased cytoskeletal stability and aberrant cell behavior on the stiff substrate, suggesting that ITGB4 could well respond to changes in physical properties of ECM. Since tissue ECM remodeling also occurs in a variety of other diseases, such as pulmonary fibrosis, acute respiratory distress syndrome and tumor cell metastasis, the findings of this study may advance the understanding and help the development of therapy for these diseases.

## 4 Materials and Methods

### 4.1 Animals

Animal studies were approved by the Central South University at XiangYa Animal Care and Use Committee. Control wild-type (WT) and airway epithelial cells (AECs)-specific ITGB4 conditionally knockout mice were granted free access to food and water under a 12 h light/12 h dark cycle in a temperature-controlled environment. The CCSP–rtTA^tg/-^/TetO-Cre^tg/-^/ITGB4^fl/fl^ triple transgenic mice with CCSP-rt-TA^tg/-^/TetO-Cre^tg/tg^ mice was described previously (Tang et al., 2020). Briefly, doxycycline (Dox; 1% in drinking water) was administered from E7.5 to the end of experiment to produce the CCSP/TetO-Cre mouse model with ITGB4 conditionally knocked out in their airway epithelial cells. All mice were housed and raised in accordance with the guidelines and regulations of our hospital.

### 4.2 Assessment of Morphology and Mechanical Properties of Lung Tissue

Lung tissue was prepared according to the method described previously (Brown et al., 2013). Briefly, the lungs were inflated by using 2% ultra-low-melting temperature agarose at 37°C, and were subsequently allowed to solidify on ice. The left lobe was dissected and the slices were generated by using a cryostatic microtome (Leica Microsystems GmbH). The tissue section has a uniform thickness of 10 um. To prevent the mechanical properties of the tissue from undue damage, anti-peeling slides were used to fix the tissue. Lung tissue morphology and mechanical properties were characterized at nanometer scale by using a Nanowizard II atomic force microscope (AFM, JPK Instruments AG, Berlin, Germany) mounted on an Olympus IX 81 inverted light microscope. The AFM tip was pyramid in shape.

The region of interest for scanning were selected by following the histological assignments for the same specimen, consistent with the view in the built-in optical microscope of the AFM instrument. To quantify the changes in the mechanical properties of ECM, stiffness and roughness parameterization was used for visualization. Cantilever spring constants were determined by using the thermal resonance frequency method (the values fall in the range of 0.03 N/m). The indentation depth of constant force was used to calculate Young’s modulus. Force-indentation profiles were fitted to a Hertz model for elastic deformation between the spheres to calculate Young’s modulus,a measurement of tissue stiffness in kPa, for each point (dispersion curves for Poisson’s ratio = 0.40).

### 4.3 Gel-Based Culture System With Tunable Matrix Stiffness

PDMS (Dow Corning, NY, United States) contains two components, oligomeric base and Sylgard184.

The mixing of oligomeric base and Sylgard184 at different ratio (normal 1:45 and stiff 1:35) with stirring could yield tunable substrates. After thoroughly mixing, the mixture was cross-linked in an oven at 60°C for 24 h. The stiffness of PDMS substrate was measured by using AFM, following the same protocol of measuring lung tissue stiffness. PDMS is hydrophobic by nature and hydrophilic after plasma treatment. PDMS could be detected by contact angle measurements. The normal and stiff substrates were coated with Matrigel (0.2 mg/ml, BD).

### 4.4 Small Interfering RNA Synthesis and Transfection

ITGB4 mRNA silencing was achieved using the siRNA technique, as previously described (Liu et al., 2010b). The effective ITGB4 siRNA (Zhang et al., 2010) (5’-CAG​AAG​AUG​UGG​AUG​AGU​U-3’) and nonsense siRNA (5’-UUC​UCC​GAA​CGU​GUC​ACG​U-3’) were designed and synthesized by Guangzhou RiboBio (RiboBio Inc., Guangzhou, China). The transfections of 16HBE14o-cells with negative control siRNA and effective silencing siRNA were performed by using Lipofectamine 3,000 (Invitrogen, United States) according to the manufacturer’s instructions. The efficiency of siRNA gene silencing was measured using RT-PCR and western blotting assay.

### 4.5 Real-Time PCR Analysis

TRIzol^®^ reagent was used to extract total RNA from lung tissues and cells. RNA was reverse-transcribed into cDNA according to the manufacturer’s protocol (Fermentas, Thermo Fisher Scientific). Real-time PCR was carried out using iTaq™ universal SYBR^®^ Green Supermix (Bio-Rad Laboratories, Hercules, CA, United States) with the CFX96 Touch™ Real-Time PCR machine (Bio-Rad). The results were calculated and normalized to the expression of GAPDH as house-keeping gene. The primers for real-time PCR were described in Table 1. The expression levels were determined by using the 2^−ΔΔCt^ method.

**TABLE 1:** Sequences of forward and reverse primers of selected genes designed for RT-PCR.

Gen		Primer sequence
Col1A1	Forward	5′-GCT​CCT​CTT​AGG​GGC​CAC​T-3′
	Reverse	5′-CCA​CGT​CTC​ACC​ATT​GGG​G-3′
Col3A1	Forward	5′-CTG​TAA​CAT​GGA​AAC​TGG​GGA​AA-3′
	Reverse	5′-CCA​TAG​CTG​AAC​TGA​AAA​CCA​CC-3′
Col4A1	Forward	5′-CTG​GCA​CAA​AAG​GGA​CGA​G-3′
	Reverse	5′-ACG​TGG​CCG​AGA​ATT​TCA​CC-3′
Col6A1	Forward	5′-CTG​CTG​CTA​CAA​GCC​TGC​T-3′
	Reverse	5′-CCC​CAT​AAG​GTT​TCA​GCC​TCA-3′
ELN	Forward	5′-CTC​TTG​TTT​CCT​TGC​CCT​GT-3′
	Reverse	5′-GCT​TCG​GAT​TGT​CTC​CCA​TTT-3′
lox	Forward	5′-TCT​TCT​GCT​GCG​TGA​CAA​CC-3′
	Reverse	5′-GAG​AAA​CCA​GCT​TGG​AAC​CAG-3′
loxl1	Forward	5′-GAG​TGC​TAT​TGC​GCT​TCC​C -3′
	Reverse	5′-GGT​TGC​CGA​AGT​CAC​AGG-3′
loxl2	Forward	5′-ATT​AAC​CCC​AAC​TAT​GAA​GTG​CC -3′
	Reverse	5′-CTG​TCT​CCT​CAC​TGA​AGG​CTC -3′
loxl3	Forward	5′-CTA​CTG​CTG​CTA​CAC​TGT​CTG​T-3′
	Reverse	5′-GAC​CTT​CAT​AGG​GCT​TTC​TAG​GA-3′
loxl4	Forward	5′-GCC​AAC​GGA​CAG​ACC​AGA​G-3′
	Reverse	5′-CCA​GGT​CAA​GGC​TGA​CTC​AAA-3′
integrin beta1	Forward	5′-TGT​GGG​CAA​CAC​TTT​GAC​CC-3′
	Reverse	5′-CAC​AGT​ACA​GCC​CTT​GAT​GTT​TA-3′
integrin alpha6	Forward	5′-TGC​AGA​GGG​CGA​ACA​GAA​C-3′
	Reverse	5′-GCA​CAC​GTC​ACC​ACT​TTG​C-3′
ITGB4	Forward	5′-GCA​GAC​GAA​GTT​CCG​ACA​G-3′
	Reverse	5′-GCA​GAC​GAA​GTT​CCG​ACA​G-3′

### 4.6 Lung Histology and Immunohistochemical Staining

Paraffin-embedded lung sections were stained with hematoxylin and eosin (H&E). Immunohistochemical staining was performed to detect the expression of COL VI, elastin and LOXL4 in lung paraffin sections by using the following antibodies: anti-collagen VI antibody (1:200, Abcam, ab6588), anti-elastin antibody (1:200, Affinity, AF5226) and anti-LOXL4 antibody (1;200, Affinity, DF13661). Zeiss Discovery.V8 Stereo microscopes (Carl Zeiss MicroImaging GmbH) and Axio-Cam ICc3 camera (Spectra Service) were used to take photos. Zeiss AxioVision Rel. 4.7 software was used for image analysis.

### 4.7 Western Blot Analysis

The total proteins were extracted from the cells using RIPA lysis buffer (Thermo Scientific, United States) containing 1% phenylmethanesulfonyl fluoride on ice. The protein concentration was measured using a BCA protein detection kit (Takara, Tokyo, Japan, T9300A). Lysates (50 μg) were separated by 10% sodium dodecyl sulfate-polyacrylamide gel electrophoresis and then transferred onto a polyvinylidene fluoride membrane, followed by blocking with 5% bovine serum albumin (BSA). The membranes were incubated along with the following primary antibodies: *β*-actin (Sigma-Aldrich, St Louis, MO, United States, A5441); Anti-ITGB4 antibody (1:1,000, Abcam, ab182120). Afterwards, the membranes were washed three times with TBST (TBS 0.1% Tween-20) and incubated with the HRP-conjugated secondary antibody (ab205718; Abcam) for 1 h at room temperature. GADPH was used as the loading control.

### 4.8 Immunofluorescence

The lobe of the left lungs was fixed in 4% paraformaldehyde, and processed for paraffin embedding. Immunofluorescent staining was performed in lung paraffin sections by using the following antibody: ITGB4 (1:200, Abcam, ab182120); CCSP (1:200, Santa Cruz, sc-365992); Anti-collagen VI antibody (1:200, Abcam, ab6588); Anti-elastin antibody (1:200, Affinity, AF5226) and anti-LOXL4 antibody (1;200, Affinity, DF13661). Immunofluorescent staining was performed in airway epithelial cells which were fixed with 4% paraformaldehyde for 15 min at room temperature. Then, the cells were washed twice, and permeabilized with 0.3% Triton X-100 in PBS for 5 min. F-actin was stained with phalloidin (1:200, tetramethylrhodamine isothiocyanate-phalloidin, dilution, Yeasen Biotechnology Co. Ltd., Shanghai, China) for 30 min at room temperature, and cells were incubated with vimentin antibody (1:200, Abcam, ab8978) overnight. The nuclei were stained with 4’,6-diamidino-2-phenylindole (Sigma-Aldrich) for 2 min. The images were acquired using a Zeiss LSM710confocal microscope (Carl Zeiss).

### 4.9 EdU Incorporation and Staining

BeyoClick™ EdU Cell Proliferation Kit with Alexa Fluor 488(Beyotime, C0071S). Cells were incubated with 50 μM EdU for 2 h for labeling. Then, 1 ml fixing solution was added at room temperature for 15 min, followed by incubation with the permeable solution. Click reaction solution was added into the cells for incubation in the dark at room temperature for 30 min. Subsequently, the reaction solution was removed and the cells were washed with detergent solution for three times, 3–5 min each time. Finally, the fluorescence test was performed.

### 4.10 Flow Cytometric Analysis

Flow cytometry was performed on a FACS Aria II flow cytometer (BD Accuri C6) with two laser channels: 488 and 635 nm. The data were analyzed by using FlowJo software (TreeStar, Ashland, OR, United States).

#### 4.11 Live-Cell Imaging and Wound-Healing Assay

Wound healing assay was conducted inaccording to a previously described method with minor modifications (Huang et al., 2020). Briefly, the human airway epithelial cells were starved for 12 h after reaching a 100% confluence. Then, the cells were passaged on normal and stiff substrates for 48 h, and transferred to the 6-well plates coated with Matrigel. In the RhoA agonist intervention groups, ITGB4−/− and NC cells cultured on the normal and stiff substrates were pre-incubated with 1 μg/ml CN03, a RhoA agonist (Cat. # CN03), for 3 h before scratch wound treatment to trigger RhoA activation. Cell monolayers were scratched with a P200 pipette tip. The images of the scratch were observed with real-time tracking and examined with an automated time-lapse microscope (Cell Observer System, Zeiss, Göttingen, Germany) equipped with a temperature and CO2 control chamber. The remaining wound closure, and the rate and trajectory of migration were analyzed using ImageJ software (Image-Pro Plus, Version 7.0) and MATLAB (The MathWorks).

#### 4.12 FRET

RhoA FRET biosensor is a gift from Professor Klaus Hahn at the University of North Carolina, which was previously described (Pertz et al., 2006).Briefly in work mechanism, the biosensor consists of a Rho-binding domain of the effector rhotekin (RBD), which specifically binds to GTP-RhoA, cyan fluorescent protein (CFP), an unstructured linker of optimized length, yellow fluorescent protein (YFP) and full-length RhoA. After the biosensor was activated by GTP-loading, and the RBD binds to Rho, the relative orientation of the two fluorophores was modified to increase FRET efficiency. Given that the fluorescent proteins were attached to one another, the FRET/CFP emission ratio at a given subcellular location could be approximately simplified as being proportional to the RhoA activation. After transfection with RhoA biosensor for 36–48 h, the cells were detached from the normal and stiff substrates with gentle Accutase digestion solution, and then seeded on fibronectin-coated 15 mm glass-bottom cell culture dish (801002, NEST, China) for 3–6 h. During the imaging process, the cells were maintained in 5% CO2 without serum at 37°C. The images were obtained by a Cell Observer System (Zeiss) equipped with the following filters (excitation, dichronic, emission): YFP (426/20 nm; 455 nm; 520/30 nm), CFP (424/24 nm, 455 nm,460/40 nm). The emission ratio was generated and computed by using the FluoCell software package in MatLab to represent the FRET efficiency before being quantified by using Prism.

## 5 Statistical Analysis

Statistical analysis was performed by using Prism 5.01 (Graph-Pad Software, San Diego California, United States). Data were presented as mean ± SD, and Student’s t-test (two-tailed) was used to evaluate inter-group differences. For multiple group comparison, data were evaluated by using one-way analysis of variance (ANOVA), followed by Bonferroni test for *post hoc* analysis. Significant difference was considered at *p* value <0.05 based on three or more independent experiments.

## Data Availability

The datasets presented in this study can be found in online repositories. The names of the repository/repositories and accession number(s) can be found in the article/supplementary material.
